# Stochastic Epigenetic Modification and Evolution of Sex Determination in Vertebrates

**DOI:** 10.1007/s00239-024-10213-9

**Published:** 2024-11-20

**Authors:** Sergio Branciamore, Andrei S. Rodin, Arthur D. Riggs

**Affiliations:** 1 Department of Computational and Quantitative Medicine, Beckman Research Institute of City of Hope, Duarte, USA; 2 Diabetes and Metabolism Research Institute, Beckman Research Institute of City of Hope, Duarte , USA

**Keywords:** Evolution of sex determination, Sex determination in vertebrates, Heteromorphic sex chromosomes, Stochastic epigenetic modification

## Abstract

In this report, we propose a novel mathematical model of the origin and evolution of sex determination in vertebrates that is based on the stochastic epigenetic modification (SEM) mechanism. We have previously shown that SEM, with rates consistent with experimental observation, can both increase the rate of gene fixation and decrease pseudogenization, thus dramatically improving the efficacy of evolution. Here, we present a conjectural model of the origin and evolution of sex determination wherein the SEM mechanism alone is sufficient to parsimoniously trigger and guide the evolution of heteromorphic sex chromosomes from the initial homomorphic chromosome configuration, without presupposing any allele frequency differences. Under this theoretical model, the SEM mechanism (i) predated vertebrate sex determination origins and evolution, (ii) has been conveniently and parsimoniously co-opted by the vertebrate sex determination systems during the evolutionary transitioning to the extant vertebrate sex determination, likely acting "on top" of these systems, and (iii) continues existing, alongside all known vertebrate sex determination systems, as a universal pan-vertebrate sex determination modulation mechanism.

**Supplementary Information:**

The online version contains supplementary material available at 10.1007/s00239-024-10213-9.

## Introduction

It is becoming increasingly clear that stochastic epigenetic changes in the early embryo are a significant aspect of development (Branciamore et al. 2018; Marion-Poll et al. 2021; Ng et al. 2018). X chromosome inactivation, wherein on a cell-by-cell basis either the paternal or maternal X chromosome is randomly chosen for inactivation, is a classic example (Ohlsson et al. 2001). There is also evidence for numerous autosomal genes involved in development showing random monoallelic expression (Branciamore et al. 2018; Marion-Poll et al. 2021; Savova et al. 2016). The impact of these types of stochastic epigenetic changes on evolution has only begun to be unraveled (Branciamore et al. 2014, 2015; Savova et al. 2016; Branciamore et al. 2018). In this context, Branciamore, *et. al.* (2014) proposed the stochastic epigenetic modification (SEM) model, in which the stochastic epigenetic modifications during a window of opportunity in early development are followed, after the window closes, by faithful maintenance of the acquired epigenetic states for the life of the organism. SEM can both increase the rate of gene fixation and decrease pseudogenization, thus dramatically improving the efficacy of evolution and likely having significant macroevolutionary effects.

The evolution of sex determination mechanisms in vertebrates is a complex process that, although having been much studied, has been proven difficult to dissect (Stöck et al. 2021). Sex determination mechanisms in vertebrates can be either genetic or environmental, sometimes both. Genetic sex determination is prevalent; however, certain reptiles and fish species exhibit environmental sex determination, such as temperature-dependent sex determination, as well as mixed (genetic and environmental) systems (Martínez-Juárez and Moreno-Mendoza 2019; Whiteley et al. 2021). Genetic sex determination is usually associated with heteromorphic sex chromosomes, notably in various independently evolved ZZ/ZW and XX/XY chromosome systems in extant vertebrates. A prominent example is the SRY sex determination (switch) gene on the Y chromosome in humans (Kashimada and Koopman 2010).

The origin and evolution of XY chromosomes, X chromosome inactivation, and dosage compensation mechanisms have been thoroughly investigated and discussed. Recent work in the field has concentrated on the evolutionary sequence of "sexual antagonism", loss of recombination in sex chromosomes, genetic degradation of sex chromosomes, loss of entire sex chromosomes, and formation of dosage compensation mechanisms (Jeffries et al. 2021; Charlesworth 2021; Kratochvíl et al. 2021), with the latest literature (Muralidhar and Veller 2022; Lenormand and Roze 2022) exposing some of the limitations of this "classical" view (i.e., the evolutionary sequence above) on the evolution of sex chromosomes. However, our understanding of the mechanisms underlying the original symmetry breakup (followed by the evolution of the heteromorphic sex chromosomes) remains limited.

In this communication, we postulate that heteromorphic sex chromosomes in vertebrates could have evolved from a pair of autosomal chromosomes carrying a sex determination locus (SRY/SOX3 analog) that has been subjected to stochastic epigenetic control. We present a corresponding mathematical model of genetic sex determination, and discuss the evolutionary (and ongoing) interplay of genetic sex determination with temperature-dependent sex determination in reptiles.

## Results

### Basic Biological Assumptions of the Model

**Fig. 1:**
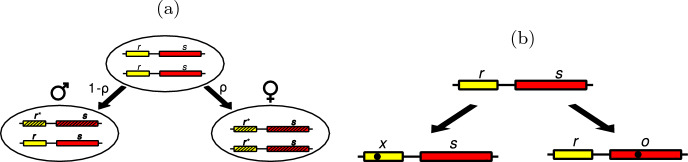
Schematic representation of the sex determination locus. The locus consists of two components: the sex determination gene, and the epigenetic modulator. We consider two possible mutations: the first one affecting the epigenetic modulator, and the second one affecting the sex determination gene. **a** The sex determination locus. r (yellow, shorter bars to the left) represents the epigenetic modulator; s (red, longer bars to the right) represents the sex determination gene. Epi-genotypes are shown in bold (in bottom ovals). An epigenetic modification ${\textbf {r}}^*$ (black-striped yellow/shorter bars) leads to the silencing of sex determination gene **s** (black-striped red/longer bars). Silencing of both regulatory elements r (with probability $\rho$) results in female (♀); silencing of one regulatory element r (with probability $1-\rho$) results in male (♂). **b** The possible mutations in the sex determination locus. Two possible mutations (black circles) lead to (on the left) inactivation of the epigenetic modulator r $\rightarrow$ x, and (on the right) function change (loss) in the gene s $\rightarrow$ o

Our mathematical model is built around the sex determination locus consisting of two components: the sex determination gene s, and the epigenetic modulator r (Fig. 1a). The epigenetic modulator can be most simply understood as the gene s promoter or, as we prefer to think, an enhancer-like element that modulates the sex determination state (on/off). Both interpretations are compatible with the mathematical modeling treatment detailed below; however, the enhancer-like element can control/influence more than one gene, which has important ramifications for subsequent sex determination system evolution. In a broad sense, the epigenetic modulator r encapsulates (for the mathematical treatment simplicity) the full spectrum of the acting epigenetic regulators in a given system—such as, for example, the histone demethylase JMJD1A that directly controls SRY expression in mammals (Kuroki et al. 2013) or the MHM1/MHM2 male hypermethylated regions directly affecting Z-linked genes expression in birds (Sun et al. 2019). It should be underlined, however, that r is an abstraction, and does not correspond to any specific epigenetic modulator identified so far in such systems.

The sex determination gene s is dominant, and its expression in the embryo leads to the male phenotype. During a window of opportunity early in development, the epigenetic modulator r can, with a probability ($\rho$), silence the sex determination gene s. In each generation, the epigentic markers are erased; therefore, although we advocate for the crucial role that epigentic modification plays in the evolution of sex chromosomes, we do not assume any direct trans-generational epigentic memory.

### Model Structure

The linear-response mathematical model, following the above assumptions, describes how stochastic epigenetic modification could have shaped the evolutionary transformation of autosomal chromosomes (carrying the sex determination gene) into proto-sex chromosomes. For clarity, all possible genotypes and associated frequency symbols are reported in Table 1. The genotypes after the epigenetic "decision" (epi-genotypes) are shown in bold throughout the text. We assume a "counting mechanism" for the epigenetic modulator (by analogy with, for example, one of the two *X* chromosomes being silenced by epigenetic mechanisms in humans): if more than one copy of the epigenetic modulator is present in the organism, then one copy is silenced at random, and the second one is silenced with the probability $\rho$.

Consider the wild type genotype rs/rs. Here, two epi-genotypes can be obtained: (1) ${\textbf {rs}}^*/{\textbf {rs}}^*$ (female ♀) with probability $\rho$ and (2) **rs**$*$/**rs** (male ♂) with probability $1-\rho$, where $*$ marks a silenced locus (Fig. 1a). We will trace the evolution of this locus while taking into consideration two possible mutations, one affecting the epigenetic modulator, and another affecting the sex determination gene (Figure  1b).

### Stability of xs Locus

*Under the model assumptions, the locus xs* *associated with any mutation r*$\rightarrow$*x that compromises the functionality of the gene regulator will spread in the population if the probability of epigenetic silencing *$\rho > 0.5$, *in parallel with the re-equilibration of the sex ratio in the population.*

Consider first the mutant x (r$\rightarrow$x) that is associated with the loss of the ability to be "counted" and is unable to work as a silencer of s. The new genotype is rs/xs, always male, as s is dominant. (Note that the homozygote xs/xs is not considered, because it is a product of the illegitimate cross between the two males rs/xs, and its appearance due to a spontaneous mutation at the same locus is vanishingly rare.)

In a random mating sexual population, let *q* stand for the frequency of the genotype rs/xs, and $1-q$, the frequency of the genotypes rs/rs. We can now compute, for each genotype, the expected population probability of all possible epi-genotypes (Table 2a). Moreover, we can also compute the probability of getting each genotype from all the possible crosses (Table 2b).

**Table 1:** Possible genotypes and associated frequency symbols

Genotype	Frequency symbol	Description
rs/rs	*z*	Original genotype
rs/xs	*q*	Non-functional regulatory element (x) in one locus
rs/ro	*k*	Non-functional sex determination gene (o) in one locus
ro/ro	*w*	Non-functional sex determination gene (o) in two loci
ro/xs	*g*	Non-functional sex determination gene (o) in one locus; non-functional regulatory element (x) in the other locus

**Table 2:** (a) The genotype/epi-genotype map and (b) possible crosses

(a)			
Genotype	Epi-genotype	Frequency in the population	Sex
rs/rs	${\textbf {rs}}^*$/**rs**	$(1-\rho )(1-q)$	♂
	${\textbf {rs}}^*/{\textbf {rs}}^*$	$\rho (1- q)$	♀
rs/xs	**rs**/**xs**	*q*	♂

(b)				
**♀ /♂**	${rs}^*$**/rs**		**rs/xs**	
${\textbf {rs}}^*/\hbox {rs}^*$	rs/rs	(1)	rs/rs	$(\frac{1}{2})$
			rs/xs	($\frac{1}{2})$

Bold indicates Epi-genotypes

$\rho$ is the probability of epigenetic silencing, *q* is the frequency of the genotype rs/xs in the population. The expected frequencies of the genotypes for each cross are shown in parentheses

Furthermore, all female epi-genotypes ${\textbf {rs}}^*/{\textbf {rs}}^*$ have genotype rs/rs, whereas male epi-phenotypes ${\textbf {rs}}^*$/**rs** and **rs**/**xs** can arise from genotype rs/rs (with probability $1-\rho$ ) and from genotype rs/xs, respectively. We are interested in the increase in the frequency of the allele xs that is present only in the genotype rs/xs (*q*). Because the regulator r is mutated, the genotype rs/xs will always produce males (Table 2b). Therefore, in this case, the change in frequency for rs/xs depends on the probability of being selected for reproduction in the male population. Under the assumption that there is no selective advantage of **rs/xs** over rs*/rs, the expected probability of selecting a male with the genotype rs/xs from the pool of all possible male genotypes for reproduction during the next generation is given by

1 $$\begin{aligned} q_{t+1}=\frac{1}{2}\frac{q_t}{q_t+(1-\rho )(1-q_t)}=\frac{1}{2}\frac{q_t}{1-\rho (1-q_t),} \end{aligned}$$

where subscript *t* emphasizes that *q* is time-dependent. Note also that here $q=1-z$ because only the r/x locus is considered. The expected frequency variation of the genotype *rs*/*xs* over time is

2 $$\begin{aligned} \Delta q_{t}=\frac{1}{2}\frac{q_t}{1-\rho (1-q_t)}-q_t=\frac{q_t[1-2\rho (1-q_t)]}{2[1-\rho (1-q_t)]}. \end{aligned}$$

The stationary state of the system can be obtained by setting $\Delta q=0$ and solving Eq. 2. There are two solutions—one trivial ($q_1=0$), and the other

3 $$\begin{aligned} q_{2}=1-\frac{1}{2\rho }. \end{aligned}$$

**Fig. 2:**
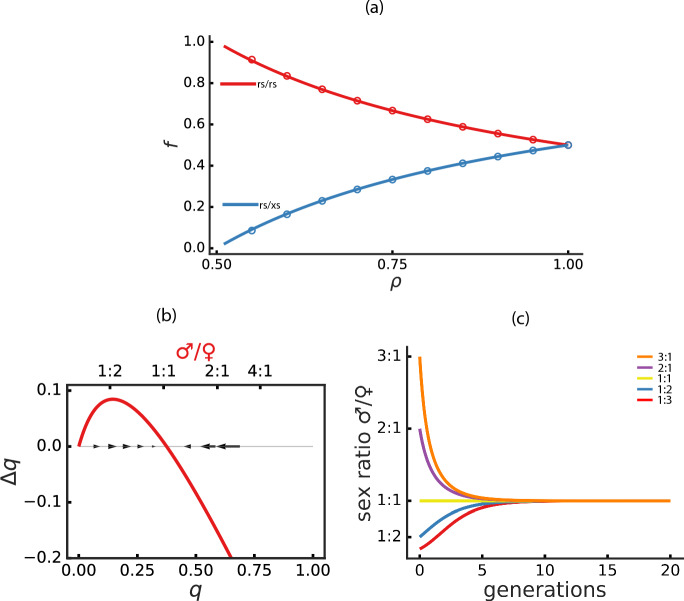
Stability of xs locus. **a** Stationary state genotype frequencies of rs/rs (*z*) in red (top) and rs/xs (*q*) in blue (bottom). Solid lines represent analytical estimates and open circles represent simulation averages. **b** Phase space representation for $\rho =0.9$ showing the variation of *q* and corresponding sex ratios. Black arrows indicate the direction of *q* variation. **c** Sex ratio dynamics for $\rho =0.8$ with various initial ratios (from top to bottom): 3:1 (orange), 2:1 (purple), 1:1 (yellow), 1:2 (blue), 1:3 (red)

We observe that the population can preserve both loci rs and xs only if the probability of epigenetic silencing $\rho$ is higher than 0.5. The new locus will show a frequency-dependent advantage over the original locus. The system will converge to $1-\frac{1}{2\rho }$ with $\Delta q > 0$ between $0<q<1-1/2\rho$ and $\Delta q < 0$ for $q>1-1/2\rho$ only for $\rho >0.5$. When $\rho <0.5$, $q_2$ is unstable, and therefore the new locus will tend to be purged from the population. We also notice that this effect is independent of the population size, because the location and the stability of the attractor depend only on $\rho$. This implies that the long-term evolutionary stable equilibrium is influenced only by the probability of epigenetic modification of the regulatory element, and not by other factors such as population size or initial sex ratio. Figure 2a depicts a typical simulation where a single copy of the mutated locus xs is introduced in the original population ($N = 1000$, $\rho =0.9$). From the simulation, we observe that the system quickly reaches the new stationary state predicted by Eq. 3. Simulations ($N=1000$) for different values of $\rho$ ($0.5<\rho \le 1$) under this scenario are in full agreement with the analytical result as in Eq. 3, and are shown in Fig. 2a. Taken together, this implies that if a mutation xs appears in the population, its frequency will increase and reach a stationary state at a value estimated by Eq. 2 that depends solely on the probability of epigenetic silencing.

It should also be noted that the dynamics of the sex ratio (♂/♀) in the population reveal the underlying evolutionary forces acting in the system. From Eq. 1, Fig. 2b, c show how the system tends to converge to a stable sex ratio of 1:1. This, unsurprisingly, is in concordance with the fundamental Fisher’s principle, and is similar to other models in the literature (Bachtrog et al. 2014). For values of $\rho >0.5$, the initial population (when only rs locus is present) has an excess of females; therefore, the new mutant locus (rs$\rightarrow$ xs) that always generates, in $\rho$-independent fashion, males (rs/xs) possesses a selective advantage over rs/rs, thus spreading in the population and re-equilibrating the sex ratio.

### Stability of ro Locus

*The locus ro* *associated with any mutation x*$\rightarrow$*o that compromises the functionality of the sex determination gene will spread in the population if the probability of epigenetic silencing *$\rho < 0.5$, *in parallel with the re-equilibration of the sex ratio in the population.*

The mutation rs $\rightarrow$ ro (Fig. 1b) leads to the loss of sex determination function. We now have three genotypes in the population: rs/ro, ro/ro and the original rs/rs with frequencies *k*, *w* and $z=1-(k+w)$, respectively. Tables 3a and 3b list the probabilities of observing each epi-genotype in the population, and the chances of getting each genotype from all the possible crosses, respectively.

**Table 3:** (a) The genotype / epi-genotype map and (b) possible crosses

(a)			
Genotype	Epi-genotype	Frequencies in the population	Sex
rs/rs	${\textbf {rs}}^*$ / **rs**	$(1-\rho )(1-(k+w))$	♂
	${\textbf {rs}}^*$ / ${\textbf {rs}}^*$	$\rho (1- (k+w))$	♀
rs/ro	**rs**/${\textbf {ro}}^*$	$(1-\rho )k/2$	♂
	${\textbf {rs}}^*$/**ro**	$(1-\rho )k/2$	♀
	${\textbf {rs}}^*/{\textbf {ro}}^*$	$\rho k$	♀
ro/ro	**ro**/${\textbf {ro}}^*$	$(1-\rho )w$	♀
	${\textbf {ro}}^*/{\textbf {ro}}^*$	$\rho w$	♀

(b)				
♀ /♂	${rs}^*$/rs		${rs/ro}^*$	
${rs}^*/rs^*$	*rs*/*rs*	(1)	*rs*/*rs*	$(\frac{1}{2})$
			*rs*/*ro*	($\frac{1}{2})$
${rs}^*$**/ro**	*rs*/*rs*	$(\frac{1}{2})$	*rs*/*rs*	$(\frac{1}{4})$
	*rs*/*ro*	$(\frac{1}{2})$	*rs*/*ro*	($\frac{1}{2}$)
			*ro*/*ro*	($\frac{1}{4}$)
${rs}^*{/ro}^*$	*rs*/*rs*	$(\frac{1}{2})$	*rs*/*rs*	$(\frac{1}{4})$
	*rs*/*ro*	$(\frac{1}{2})$	*rs*/*ro*	($\frac{1}{2}$)
			*ro*/*ro*	($\frac{1}{4}$)
${ro}^*$**/ro**	*rs*/*ro*	(1)	*rs*/*ro*	$(\frac{1}{2})$
			*ro*/*ro*	$(\frac{1}{2})$
${ro}^*{/ro}^*$	*rs*/*ro*	(1)	*rs*/*ro*	$(\frac{1}{2})$
			*ro*/*ro*	$(\frac{1}{2})$

Bold indicates Epi-genotypes

$\rho$ is the probability of epigenetic silencing, *q* is the frequency of the genotype rs/xs in the population. The expected frequencies of the genotypes for each cross are shown in parenthesis

In order to evaluate the probability for each epi-genotype to be selected for reproduction, we need to compute the total frequencies of females and males in the population (Eqs. 4 and 5), and then relative probabilities for an epi-genotype to be selected as either male or female:

4 $$\begin{aligned} Z_f= & \sum _{i \in F} {P_i}=\frac{2\rho + \left( 1 - \rho \right) \left( p + 2 w\right) }{2}, \end{aligned}$$

5 $$\begin{aligned} Z_m= & \sum _{i \in M} {P_i} = \frac{\left( 1-\rho \right) \left( 2-p - 2 w\right) }{2}, \end{aligned}$$

where $P_i$ is the probability of the epi-genotype in F or M set of all possible female and male epi-genotypes, respectively:

6 $$\begin{aligned} F= & \{{\textbf {rs}}^*/{\textbf {rs}}^*, {\textbf {rs}}^*/{\textbf {ro}}, {\textbf {rs}}^*/{\textbf {ro}}, {\textbf {rs}}^*/{\textbf {ro}}^*, {\textbf {ro}}/{\textbf {ro}}^*, {\textbf {ro}}^*/{\textbf {ro}}^*\}, \end{aligned}$$

7 $$\begin{aligned} M= & \{{\textbf {rs}}^*/{\textbf {rs}},\quad {\textbf {rs}}/{\textbf {ro}}^*\} \end{aligned}$$

Using the above expression, we can estimate the expected genotype frequencies in the next generation for the genotypes rs/ro and ro/ro and obtain the non-trivial analytical solutions for *k* and *w* for the system that will converge to a sex ratio equal to 0.5 (see Supplemental Information for detailed derivations):

8 $$\begin{aligned} w_1&= \frac{1+2\rho ^{3} - 3 \rho ^{2} - \sqrt{2 (1-\rho )^{3}}}{2(\rho -1)^{3}}, \nonumber \\ w_2&= \frac{1+2\rho ^{3} - 3 \rho ^{2} + \sqrt{2 (1-\rho )^{3}}}{2(\rho -1)^{3}}, \end{aligned}$$

9 $$\begin{aligned} k_1&= \frac{2 + 2 \rho ^{2} - 4 \rho - \sqrt{ 2(1-\rho )^3}}{(1-\rho )^3}, \nonumber \\ k_2&= \frac{2 + 2 \rho ^{2} - 4 \rho + \sqrt{2(1-\rho )^3}}{(1-\rho )^3.} \end{aligned}$$

Finally, we observe that the derived solutions for the model are acceptable only if the frequencies are in the range [0, 1] which holds only for $k_1$, $w_1$ and for $0 \le \rho < 0.5$. Notably, the system has no solution for sufficiently large $\rho$, and therefore in such case the locus ro cannot be preserved. In order to evaluate the critical "cutoff" value of $\rho$ for which the locus ro can still be preserved, we have performed the stability analysis of the above system at 0. Specifically, we dissected the behavior of the dynamical system close to the solution, in order to ascertain whether a small perturbation would "circle back" to the solution, or whether the population will be attracted to another solution. In order to do that we need to evaluate the Jacobian matrix, which at *O* is

10 $$\begin{aligned} J(0,0)&= \left[ \begin{array}{ll}\frac{1 - 2 \rho }{4 \rho } & \frac{1}{\rho }\\ 0 & -1\end{array}\right] ,&\lambda ^{2} + \frac{6 \rho - 1}{4 \rho } \lambda + \frac{2 \rho - 1}{4 \rho }&= 0. \end{aligned}$$

The solutions of the above equation are the eigenvalues of the Jacobian, $\lambda _1=-1$ and $\lambda _2=(1 - 2\rho )/4\rho$. The point *O* is asymptotically stable if both eigenvalues are negative, which implies $\rho >1/2$. Therefore, if $\rho >1/2$, then the system tends to preserve the original genotype rs/rs and purge out the homozygote ro/ro and heterozygote rs/ro. On the other hand, if $\rho <1/2$, the system evolves towards the stable point that can be computed using Eqs. 8 and  9.

**Fig. 3:**
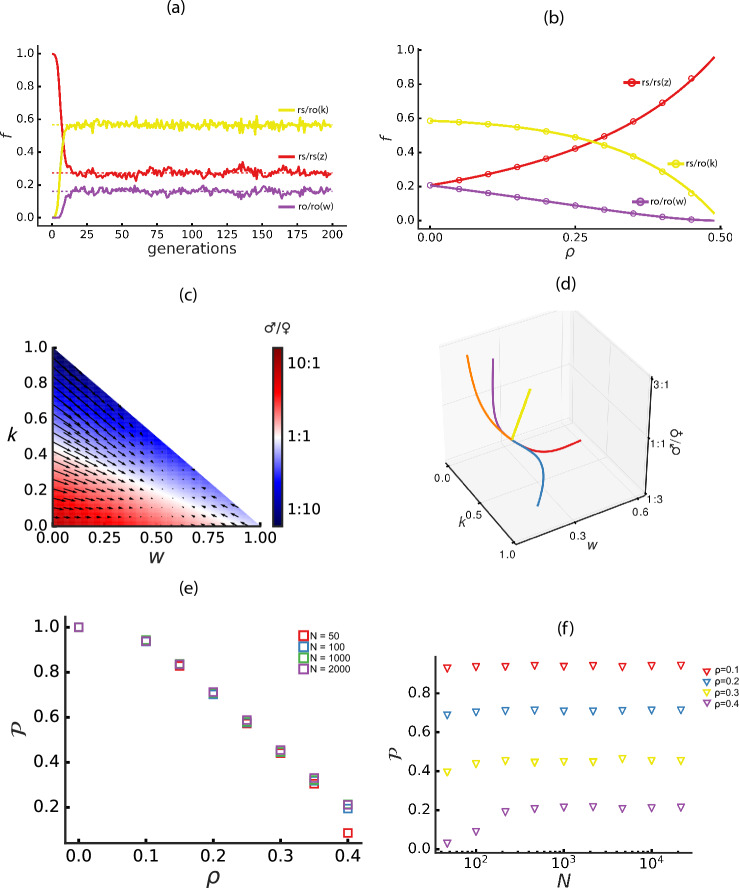
Stability of ro locus. **a**, **b** Observed frequencies for the genotypes: rs/rs (*z*), initialized at $1-\frac{1}{2N}$, red; rs/ro (*k*), initialized at $\frac{1}{2N}$, yellow; ro/ro (*w*), initialized at zero, purple. **a** Simulation example with *N*=1,000 and $\rho$=0.1. **b** Analytically derived stationary states (solid lines) with simulation results (circles). **c** Phase space representation of genotype frequencies *k* and *w*. The color gradient shows sex ratio variation, with black arrows indicating the direction of changes and pointing to the stable attractor at the 1:1 sex ratio. **d** Trajectories of individual population dynamics for given initial conditions of *k* and *w*. All trajectories converge to an attractor with a 1:1 sex ratio. **e** Probability (${\mathcal {P}}$) of reaching the stationary state as a function of $\rho$, represented by squares for different *N* values. (f) ${\mathcal {P}}$ as a function of *N* (in $log_{10}$ scale), represented by triangles for different $\rho$ values. Each data point represents an average of $n\le 10,000$ simulations

Only under the above conditions would the new locus be preserved in the population. The analytical solutions of the corresponding equations, initial frequencies, and the simulation results for different values of $\rho$ and *N* are depicted in Figs. 3a and 3b.

Similarly to the xs locus fixation, in this case the evolutionary forces also drive the tendency towards re-equilibration of the sex ratios in the population. For low values of $\rho$ ($<0.5$), the population sex ratio is biased towards males. The mutant locus ro is female ($\rho$-independent) when homozygous, thus allowing the population to converge to a sex ratio 1:1 (as shown in the phase diagram representation in Fig. 3c) independently of the initial sex ratio (Fig. 3d).

We conclude by estimating the probability of reaching the stationary state (${\mathcal {P}}$) following Eqs. 8 and 9. When $\rho$ is low ($<0.4$) the sex ratio is strongly biased towards males and $\rho$ has a selective advantage that linearly increases as $\rho$ decreases. This selective advantage dominates the drift effect so that for sufficiently large populations ($N>100$), ${\mathcal {P}}$ is almost entirely independent of population size (Fig. 3e, f. This phenomenon is not compatible with what is expected under neutral evolution, where the probability of fixation should be proportional to the initial frequency in the population, which in this case is $\frac{1}{2N}$. On the other hand, when $\rho$ is close to 0, this indicates a deviation from the optimal sex ratio within the system. Consequently, this increases the selective advantage of ro and, therefore, the probability of fixation.

### Further Evolution of the Sex Determination Locus

Once one of the two new alleles (either xs or ro) is fixed in the population, the second one will be fixed at a rate and with a probability that are consistent with neutral evolution, independently of $\rho$ and the population size *N*.

**Fig. 4:**
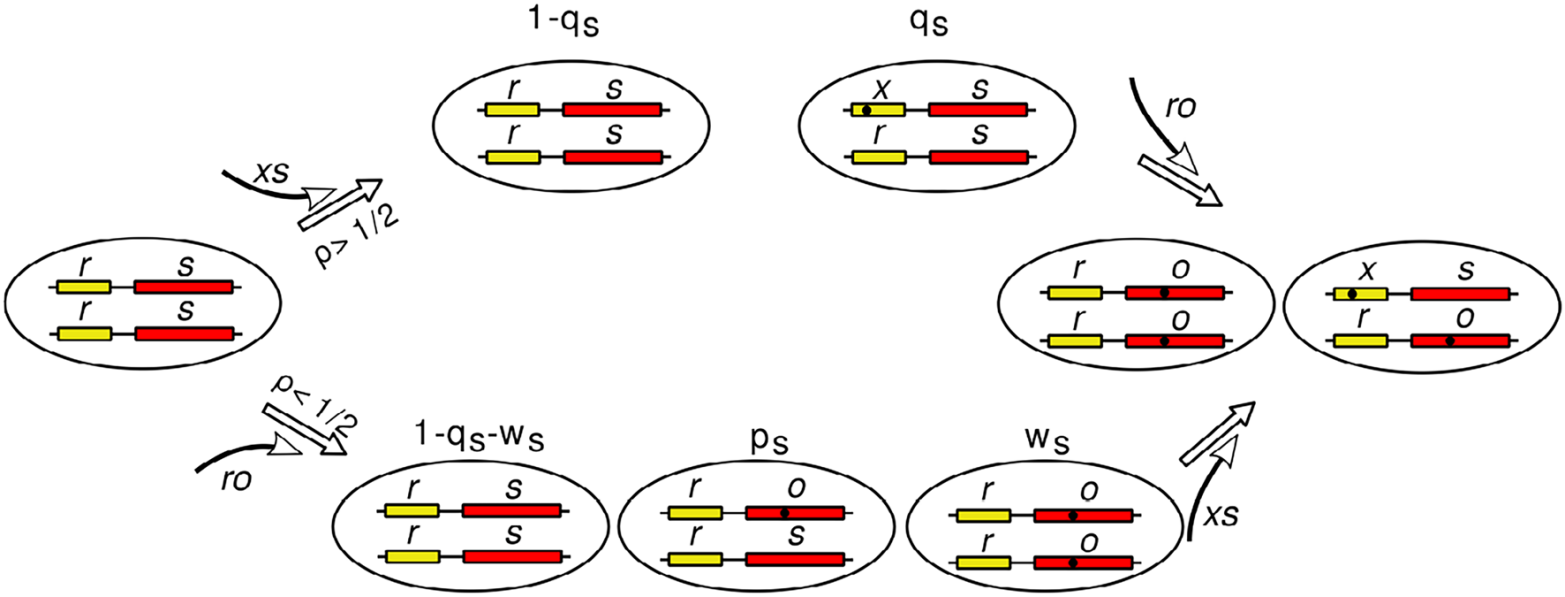
Schematic representation of the SD evolution model. From the top: (1) Stable stationary state obtained for $\rho >1/2$ when the mutant xs is introduced in the population. The expected frequencies $q_s$ of the genotype *rs*/*xs* depend on $\rho$ and are given by Eq. 3. (2) For $\rho <1/2$, when the mutant ro is introduced in the population, the genotypes ro/rs and ro/ro have the expected frequencies $k_s$ and $w_s$, respectively

From all of the above, we conclude that under the given model assumptions the original haplotype rs would most likely evolve towards the preservation of the haplotypes ro and xs for values of $\rho$ lower or higher than 1/2, respectively (Fig. 4). We are now interested in the further evolution of the system when the haplotypes rs, rx and ro are simultaneously present. The possible extant genotypes in the population are now rs/xs, rs/ro, ro/ro, ro/xs, rs/rs with associated frequencies *q*, *p*, *w*, *g*, and $z=1-(q+k,w+g)$, respectively. Just as before, we are interested in the evolution of genotype frequencies in the population. The frequencies of the possible epi-genotype as a function of $\rho$ are shown in Table 4 and Supplemental Information Table 4. The frequencies of male and female phenotypes in the population are given by

11 $$\begin{aligned} Z_{f}= & \frac{k \left( \rho + 1\right) + 2 \rho z + 2 w}{2}, \end{aligned}$$

12 $$\begin{aligned} Z_{m}= & \frac{2 g + 2 q + k \left( 1 - \rho \right) - 2 \rho z + 2 z}{2}, \end{aligned}$$

and we can compute the expected frequencies in the next generation for each genotype as shown in Supplemental Information Equation 13.

**Table 4:** Genotype/epi-phenotype map

Genotype	Epi-genotype	Frequency in the population	Sex
rs/rs	${\textbf {rs}}^*$ / **rs**	$(1-\rho )[1-(q+k+w+g)]$	♂
	${\textbf {rs}}^*$ / ${\textbf {rs}}^*$	$\rho [1- (q+k+w+g) ]$	♀
rs/xs	**rs**/**xs**	*q*	♂
ro/xs	**ro**/**xs**	*g*	♂
rs/ro	**rs**/${\textbf {ro}}^*$	$(1-\rho )k/2$	♂
	${\textbf {rs}}^*$/**ro**	$(1-\rho )k/2$	♀
	${\textbf {rs}}^*/{\textbf {ro}}^*$	$\rho k$	♀
ro/ro	**ro**/${\textbf {ro}}^*$	$(1-\rho )w$	♀
	${\textbf {ro}}^*/{\textbf {ro}}^*$	$\rho w$	♀

Bold indicates Epi-genotypes

$\rho$ is the probability of epigenetic silencing, *q*, *k*, *w*, and *g* are the frequencies in the population of the genotypes *rs*/*xs*, *rs*/*ro*, *ro*/*ro* and *ro*/*xs*, respectively

The preservation in the population of the two new haplotypes ro and xs can be interpreted as the seed for the formation of the proto-X and Y chromosomes (or other types of sex chromosomes, in non-mammalian species). The individuals with two ro haplotypes are always female, whereas organisms with one xs are male (analogous to the current XX and XY system in mammals). In addition, organisms with genotypically ambiguous sex genotypes (rs/rs and ro/rs) might still co-exist in populations. It should also be noted that while the first step of the process (Fig. 4, on the left) is comparatively swift (just a few generations), the second step (Fig. 4, on the right) takes a relatively long time (hundreds to thousands of generations), as it is essentially a random drift scenario.

**Fig. 5:**
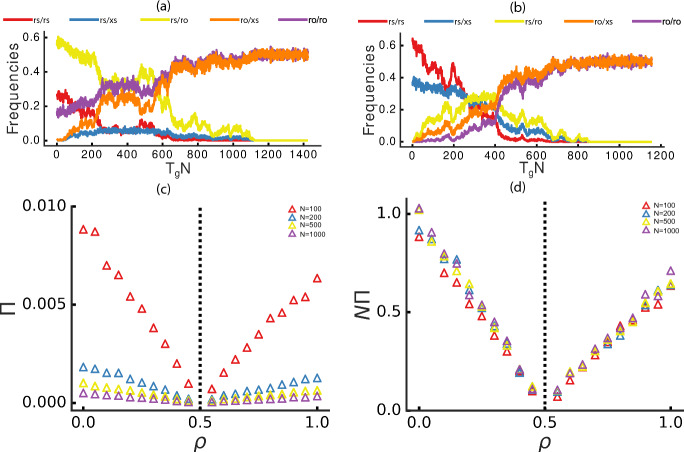
Time to (and probability of) fixation of the two haplotypes ro and xs. **a**, **b** Population dynamics for $\rho$=0.1 (**a**) and $\rho$=0.8 (**b**) with *N*=1,000. Initial frequencies are computed using Supplemental Information Eq. 8, with a single haplotype rs/ro. Observed genotype frequencies are color-coded: rs/rs (*z*) in red, rs/xs (*q*) in blue, rs/ro (*k*) in yellow, ro/xs (*g*) in orange, and ro/ro (*w*) in purple. **c** Estimated probability of fixation $\Pi$ for the proto-male ro/xs ($\rho <0.5$) and proto-female ro/ro ($\rho >0.5$), computed by solving a system of non-linear equations (Supplemental Information Eq. 13), as a function of $\rho$ for different *N* values (from top to bottom: *N*=100, red; 500, blue; 1000; yellow; 2000, purple). **d** Scaled probability $N \Pi$ as a function of $\rho$, represented by triangles for different *N* values. Each data point is an average of n$\ge 10,000$ simulations. Colors in (**d**) correspond to the same *N* values as in (**c**)

Using simulation experiments, we have estimated the probability of fixation of the two haplotypes ro and xs (Fig. 5). It appears that the probability of fixation of the haplotype ro decreases as $\rho$ increases, approaching zero as $\rho$ approaches 1/2. With $\rho$ higher than 1/2, the probability of fixation of the proto-male haplotype xs increases as $\rho$ approaches 1 (Fig. 5c). Interestingly, the probability of fixation $\Pi$ changes alongside *N* in such a fashion that their product, scaled probability $N\Pi$ (Fig. 5d) becomes largely independent of *N*, which is what one would expect for a neutral process.

### Evolution of Sex Determination Locus Under Mutation Pressure

Extending the model by introducing mutations demonstrates that the proto-X chromosome spreads quickly and remains stable within the population.

**Fig. 6:**
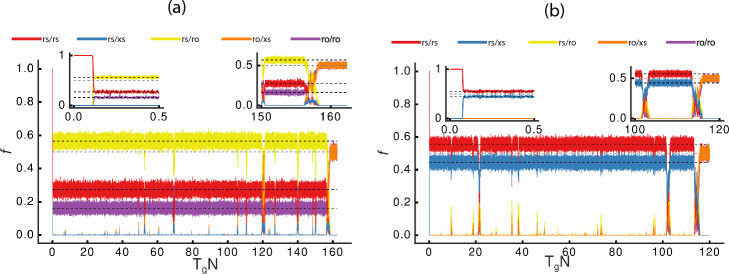
Population dynamics under mutation pressure. The system is initialized with population size *N*=1,000, and with only the haplotype rs/rs (*z*) (in red) present and subject to $\mu =10^{-5}$ mutation rate. Frequencies are computed using Supplemental Information Eq. 8, and are reported for rs/xs (*q*) in blue, rs/ro (*k*) in yellow, ro/xs (*g*) in orange, and ro/ro (*w*) in purple. Dashed black lines indicate expected frequencies for $proto-X$ ro/ro and $proto-Y$ ro/xs. The insets at the top "zoom in" the early and late stages (left and right panels, respectively), illustrating the initial stationary state and the final fixation state. **a** Probability of silencing $\rho =0.1$—dashed black lines indicate the analytically computed stationary state for rs/rs, rs/ro and ro/ro using Supplemental Information Eq. 8. **b** Probability of silencing $\rho =0.9$ —dashed black lines indicate the analytically computed stationary state for rs/rs and rs/xs using Eq. 3

We now introduce mutation pressure (allowing the loci to mutate with probability $\mu$ in each generation), and show that the system is able to evolve spontaneously from the initial haplotype rs to the proto-X ro and proto-Y xs (Fig. 4). Note that mutations in this simulation do not create any new functions—they simply compromise the functionality of either the regulatory element r$\rightarrow$ x or the sex determinant gene s $\rightarrow$ o. Two examples of such dynamics are shown for $N=1000$, $\mu =10^{-5}$ and $\rho =0.1$ (Fig. 6a) and 0.9 (Fig. 6b). In both cases, just as expected from the above analyses, the system quickly reaches a stable stationary state described by Eq. 3 and Supplemental Information Eq. 8 for $\rho >0.5$ and $\rho <0.5$, respectively (left inset in Fig. 6). As predicted, the system remains in this stable configuration for a long time, with the new mutants appearing and disappearing during the evolutionary process, until a "successful" new mutant gets rapidly fixed in the population (right inset in Fig. 6).

## Discussion

*Stochastic epigenetic phenomena and sex determination evolution.* X chromosome inactivation is a quintessential example of an epigenetic phenomenon. By analogy, it would not be unreasonable to suppose that evolution of heteromorphic sex chromosomes was also subjected to epigenetic mechanisms, as stochastic epigenetic modification (SEM) is becoming increasingly recognized as a key factor in evolution. In developing the above mathematical model linking SEM and sex chromosomes differentiation, we have put forward a set of assumptions (following our prior work on monoallelic expression (Branciamore et al. 2014): First and foremost, we assume that the sex determination dominant locus (*s*) is epigenetically controlled by a regulator (enhancer-like) locus *r* that is able to silence *s* with probability $\rho$. The epigenetic "decision" occurs only during a window of opportunity in an early development stage, when the first cell lineage determinations are being made. We also assume that this decision is subsequently maintained for the entire life of the organism. Finally, we assume that all genetic markers are erased during gametogenesis. Our model demonstrates that this set of assumptions is sufficient to drive the functional symmetry breakup and trigger the evolution of heteromorphic sex chromosomes from the initial homomorphic chromosome configuration, without presupposing any allele frequency differences. The model is highly parsimonious, in that (i) it takes advantage of the existing epigenetic mechanisms and fortuitously re-uses them in a new context, and (ii) it only employs loss-of-function mutations and does not require less probable novel gain-of-function mutations.

*Deterministic vs. stochastic models of sex determination evolution.* Previous models of sex chromosomes evolution either assumed "sexual antagonism" as the principal driving force or, more recently, questioned the "sexual antagonism" hypothesis while emphasizing gene regulation and genetic drift (Lenormand and Roze 2022). However, the emergence of the proto-XY (proto-ZW) sex chromosomes in the first place remains underexplored. Seminal work by Bull ( Bull (1983), p. 66) put forward a model containing two SD genes that, while superficially similar to our model (due to the presence of two main factors), is fundamentally distinct in that it does not allow for stochasticity, being fully deterministic (which is a narrower class of models than stochastic models). This results in different evolutionary dynamics — importantly, (i) under baseline conditions, Bull-type models can lead to infinite solutions, whereas in the stochastic model there is a unique attractor; (ii) in the stochastic model, one genotype can map to multiple sexes, allowing a degenerate correspondence between genotype and sex. In our case, genotype-phenotype mapping is modulated by epigenetic modification ("epi-phenotype"). More recently, Perrin (2016) argued for the possibly high salience of "developmental noise" (i.e., stochasticity) in the context of sex-determining factors; this broad framework, while not touching directly on epigenetic factors, complements our explicit mathematical modeling.

*Maintenance of balanced sex ratio.* Although the stochastic epigenetic mechanism is the focal point of our study, we also demonstrate, in an auxiliary line of inquiry, that early in the evolutionary trajectory the driving force behind the fixation of the *xs* and *ro* mutant alleles was the tendency to re-establish the 1:1 sex ratio in the population. This is congruent with the fundamental Fisher’s principle (Smith and Maynard-Smith 1978; Fisher 1930) and prior models in the literature (Bachtrog et al. 2014); however, it is important to underline that we did not stipulate any external selection coefficient (pushing the model towards the balanced sex ratio) in the model formulation. Rather, this is a natural tendency representing, under the model assumptions, the stable evolutionary strategy (Smith and Maynard-Smith 1978). This can be intuitively understood by observing that if there is a sex disbalance in the random mating population, the allele that is most likely to produce the rarer sex (in accordance with the probability of epigenetic modification) would have a higher chance to be selected for mating. This will increase the frequency of the rarer sex until, eventually, the balance is achieved. From that point on there will be no advantage for any allele, and the population will be evolutionary stable. In other words, the frequencies of the male and female sex in the population depend on the allele frequencies as well as on the probability of epigenetic silencing $\rho$. Our analysis demonstrates that the balanced sex ratio of 1:1 represents the fixed stable point for the system as shown in Figs. 3c, 3d — once it is reached, any minute perturbation in the allele frequencies will be absorbed, and the population will be driven back towards the 1:1 sex ratio.

*Gaining independence from the environment.* "Ohno’s paradigm" (Ohno 1967) posits that there is a tendency to move from the "haphazard", often partially or fully environment-dependent, sex determination mechanisms to more "entrenched" genetic sex determination mechanisms and sex chromosomes. In other words, as we progress along the evolutionary tree, there is a shift from the partially environmental-stochastic sex determination to fully genetic sex determination, with a rigid sex chromosomes system. This shift can be interpreted as gaining more independence from the environment (alongside such events as, for example, acquiring placenta in mammals).

*Macroevolutionary sequence of events.* Our mathematical model demonstrates how heteromorphic sex chromosomes - based genetic sex determination can be realized via SEM. We hypothesize that such SEM apparatus predated vertebrate origin and evolution, and continues existing, alongside various vertebrate sex determination systems, as a universal "pan-vertebrate" mechanism. The circumstantial evidence in favor of the above conjuncture in, for example, reptiles (and, by inference, in proto-birds and proto-mammals) is four-fold: (i) in these species, there are numerous homologs of SRY/SOX3/SF-1-like sex determination "switch" genes (Ezaz et al. 2017; Capel 2017; Wallis et al. 2008), (ii) they have large, strongly regulated promoter regions that are highly susceptible to SEM (Deveson et al. 2017; Weber et al. 2020), (iii) there is a broad, and growing, body of evidence for sex determination in reptiles (and fish species) being controlled and mediated by epigenetic regulation in the first place (Deveson et al. 2017; Ge et al. 2018; Georges and Holleley 2018; Whiteley et al. 2021; Weber and Capel 2021; Piferrer 2021; Kuroki and Tachibana 2018), and (iv) it appears that sex chromosomes-based genetic sex determination systems in many reptile lineages are quite ancient, occasionally acting concurrently with the environmental (temperature-dependent) sex determination (Whiteley et al. 2018; Rovatsos et al. 2019a, b).

Therefore, the following sequence of events appears to be plausible: at first, environmental temperature-dependent sex determination (as well as the maintenance of the balanced sex ratio) is realized through the temperature change factor directly modulating epigenetic regulation of sex determination "switch" gene promoters and other sex determination system components. Then, the temperature change factor is altered significantly (or disappears altogether — during, for example, the transition from reptiles to placental mammals). However, the existing epigenetic regulation apparatus is still there, it just does not have the temperature modulator continuously and decisively acting "on top" of it any longer. In other words, the linkage between the temperature change factor and the SEM mechanism is weakened, becoming irrelevant in the homeothermic vertebrates (mammals). Essentially, the SEM mechanism, as mathematically modeled above, is left to its own devices (a state that can be defined by the relatively constant silencing probability parameter $\rho$) — which leads to the heteromorphic sex chromosomes origination.

Such processes might have occurred independently and repeatedly across various vertebrate lineages. In this, SEM might act as a threshold-determining mechanism, dovetailing with the “threshold” model of sex determination transitions proposed in Quinn et al. (2011); Schwanz et al. (2016), although in our case the transition has occurred from environmental to genetic sex determination.

*Ongoing sex determination evolution in vertebrates.* If the environmental factors (temperature) keep fluctuating, they can continue modulating/controlling the existing epigenetic regulation machinery and thus influencing sex determination, even with the sex chromosomes already in place. Consider, for example, the curious case of an egg-laying Australian lizard, *Pogona vitticeps*, where both genetic and environmental (temperature-dependent) sex determination systems appear to interplay (Whiteley et al. 2018, 2021; Holleley et al. 2015). Our current understanding is that the sex determination systems are highly labile across various reptilian clades (Rovatsos et al. 2019a) and even occasionally within a single reptile species. We posit that sex determination evolution (along the lines of the proposed model; see Step 2 in Fig. 4, on the right) is still ongoing in the *Reptilia*, perhaps switching on and off and going back and forth following notable environmental (temperature) fluctuations.

While we do not intend here to speculate on the mechanistic underpinnings of the $\rho$ parameter ($\rho$ being just a convenient single-parameter quantitative encapsulation of all the biological processes underlying SEM), it clearly follows from our model that more "extreme" (high or low) $\rho$ values lead to the accelerated proto-sex chromosomes formation, whereas "middle-of-the-road" $\rho$ values lead to the continuing, if ultimately temporary, preservation of the status quo (such as largely temperature-dependent sex determination). It is possible, then, that more extreme $\rho$ values directly reflect sudden drastic (but stable and enduring) changes in the environment and, in turn, provoke the processes eventually leading to the accelerated development of the environment-independent rigid sex chromosomes - based sex determination. As an interesting, if not dispositive, observation, reptiles native to the harsher (for the reptiles, e.g., central European) climes tend to show fixed sex chromosomes - based sex determination systems (Rovatsos et al. 2019b). Another suggestive observation is that *Pogona vitticeps* shows no evidence of sex reversal in the more extreme (for the species) parts of their range, while there is consistent evidence for regular sex reversal in the moderate part of the range (Castelli et al. 2021).

So far, we have mostly concentrated on the reptiles-to-mammals (and, by analogy, birds) transition. However, similar processes have been (and continue) taking place in various fish clades, in which there is a wide variety of environment-dependent sex determination, labile genetic sex determination, and heteromorphic sex chromosomes - based sex determination systems, often working simultaneously and interdependently (Devlin and Nagahama 2002; Weber and Capel 2021). Therefore, the SEM model might be universal across the vertebrates, progressing independently throughout various lineages.

*Limitations and future research directions.* In our model we assume, for mathematical simplicity, that the $\rho$ parameter (and, by inference, the overall effect of the epigenetic modulator r) is constant. However, it might change in time, within a single population. If we accept that environment (temperature) can immediately affect $\rho$, then it would be worthwhile to expand our model by introducing realistic time-dependent $\rho$ modulation. Similarly, a promising future research direction would be to incorporate the existing (and growing) body of knowledge on the actual molecular underpinnings of epigenetic regulation of sex determination (Deveson et al. 2017; Ge et al. 2018; Whiteley et al. 2021; Weber and Capel 2021; Piferrer 2021; Kuroki and Tachibana 2018) into our model, specifically the $\rho$ parameter and its dynamics. Another assumption in our model is that of the sex determination gene’s dominance. While true for mammals, this assumption does not hold across the vertebrates *en toto*; therefore, one of the future research directions would be extending the model by relaxing this assumption to dosage-based sex determination. Our intuition is that the principal effect, i.e., the tendency of the population to break the original chromosome functional symmetry driven by the trend towards re-equilibration of sex balance, will be preserved.

It remains to note that, congruent with our model, stochastic epigenetic mechanisms affecting sex determination are to be found early in the evolution of sex chromosomes, and this is certainly the case for the organisms with temperature-dependent sex determination. However, to further corroborate our model, it would be essential to demonstrate stochastic epigenetic silencing playing a role in determining the sex ratio structure of the population for the organisms with the fully genetic sex determination, which is a promising future research direction (similar to our previous work on the stochastic epigenetic silencing of the autosomal genes).

*In summary,* we suggest that the SEM mechanism might have predated the origins of both the environmental sex determination and genetic sex determination, and might have been conveniently and parsimoniously co-opted by them during the evolutionary transitioning to the extant vertebrate heteromorphic sex chromosomes - based sex determination systems.

### Materials and Methods

All of the methodology (mathematical models) in this study is based on the general Wright-Fisher model, which has discrete, non-overlapping generations and fixed population size. Epigenetic modifications were introduced into the models following (Branciamore et al. 2014, 2015). In addition to the analytical treatment, numerical simulations were carried out for various modeling scenarios using the parameter ranges reported in the Results section. All the simulation were initialized with an initial frequency of the new mutant set at 1/*N*. To compute the probability ${\mathcal {P}}$ of reaching the stationary state, each simulation was labeled as "successful" and stopped if/when the predicted stationary state was reached, and "unsuccessful" if the seeded allele was lost during the simulation. All statistical estimates from simulations were obtained by using at least 10,000 random replications (unless otherwise noted in Results).

*Monte Carlo Simulation Steps*

1. Initialize the diploid population with one mutant locus xs (or rs ) and the remaining $2N-1$ loci as wild-type rs.
2. Introduce epigenetic modifications with probability $\rho$ according to the described model, and identify sex for each organism.
3. Select a male organism and a female organism from the pool of males and females with replacement, and generate one offspring as a random assortment of the parents’ genotypes after removing all epigenetic markers. If mutations are considered, they are introduced at random with probability $\mu$. Repeat this step *N* times.
4. Repeat steps 2 and 3 until the mutant locus is either fixed or lost.

The numerical simulations were carried out using Python on a high performance computing cluster.

## Supplementary Information

Below is the link to the electronic supplementary material.

Supplementary file1 (PDF 146 KB)
