# Correction: Stochastic Epigenetic Modification and Evolution of Sex Determination in Vertebrates

**DOI:** 10.1007/s00239-024-10229-1

**Published:** 2025-01-03

**Authors:** Sergio Branciamore, Andrei S. Rodin, Arthur D. Riggs

**Affiliations:** 1 Department of Computational and Quantitative Medicine, Beckman Research Institute of City of Hope, Duarte, USA; 2 Diabetes and Metabolism Research Institute, Beckman Research Institute of City of Hope, Duarte , USA

**Journal of Molecular Evolution (2024) 92:861–873** 10.1007/s00239-024-10213-9

In the original publication, both the authors Sergio Branciamore and Andrei S. Rodin should have been denoted as corresponding author.

The original article has been corrected.

