# Supplementary material for: Stochastic Epigenetic Modification and Evolution of Sex Determination in Vertebrates
Source: J Mol Evol. 2024 Nov 20;92(6):861–73. doi: 10.1007/s00239-024-10213-9 (PMC11646274; doi:10.1007/s00239-024-10213-9)
Supplement: Supplementary file 1 — Supplementary file1 (PDF 146 KB) [file 239_2024_10213_MOESM1_ESM.pdf]

Stochastic Epigenetic Modification as a Parsimonious Mechanism for Origin and Evolution of Heteromorphic Sex Chromosomes in Vertebrates (Supplemental Information)

October 1, 2024

1. Stability of ro locus

The mutation rs $\rightarrow$ ro leads to the loss of sex determination function. We have three genotypes in the population: rs/ro, ro/ro and the original rs/rs with frequencies k, w and $z = 1 - (k + w)$, respectively. Tables 1 and 2 list the probabilities of observing each epi-genotype in the population, and the chances of getting each genotype from all the possible crosses, respectively.

genotype	epi-genotype	frequencies in the population	sex
rs/rs	$\mathbf{rs}^*$ / $\mathbf{rs}$	$(1-\rho)(1-(k+w))$	♂
	$\mathbf{rs}^*$ / $\mathbf{rs}^*$	$\rho(1-(k+w))$	♀
rs/ro	$\mathbf{rs}/\mathbf{ro}^*$	$(1-\rho)k/2$	♂
	$\mathbf{rs}^*/\mathbf{ro}$	$(1-\rho)k/2$	♀
	$\mathbf{rs}^*/\mathbf{ro}^*$	ρk	♀
ro/ro	$\mathbf{ro}/\mathbf{ro}^*$	$(1-\rho)w$	♀
	$\mathbf{ro}^*/\mathbf{ro}^*$	ρw	♀

Table 1: Genotype / epi-genotype map. ρ is the probability of epigenetic silencing, r and w are the frequencies in the population of the genotypes rs/ro and ro/ro, respectively.

The total frequencies of females and males in the population (Equations 1 and 2), and the relative probabilities for an epi-genotype to be selected as either male or female:

$$Z_f = \sum_i^F P_i = \frac{2\rho + (1-\rho)(p+2w)}{2}, \tag{1}$$

$$Z_m = \sum_i^M P_i = \frac{(1-\rho)(2-p-2w)}{2}, \tag{2}$$

where M and F stand for the sets of all possible male and female epi-genotypes, respectively:

$$F = \{\mathbf{rs}^*/\mathbf{rs}^*, \mathbf{rs}^*/\mathbf{ro}, \mathbf{rs}^*/\mathbf{ro}, \mathbf{rs}^*/\mathbf{ro}^*, \mathbf{ro}/\mathbf{ro}^*, \mathbf{ro}^*/\mathbf{ro}^*\}, \tag{3}$$

♀/♂	rs*/rs		rs/ro*	
**rs*/rs***	rs/rs	(1)	rs/rs	($\frac{1}{2}$)
			rs/ro	($\frac{1}{2}$)
**rs*/ro**	rs/rs	($\frac{1}{2}$)	rs/rs	($\frac{1}{4}$)
	rs/ro	($\frac{1}{2}$)	rs/ro	($\frac{1}{2}$)
			ro/ro	($\frac{1}{4}$)
**rs*/ro***	rs/rs	($\frac{1}{2}$)	rs/rs	($\frac{1}{4}$)
	rs/ro	($\frac{1}{2}$)	rs/ro	($\frac{1}{2}$)
			ro/ro	($\frac{1}{4}$)
**ro*/ro**	rs/ro	(1)	rs/ro	($\frac{1}{2}$)
			ro/ro	($\frac{1}{2}$)
**ro*/ro***	rs/ro	(1)	rs/ro	($\frac{1}{2}$)
			ro/ro	($\frac{1}{2}$)

Table 2: List of all possible crosses. The expected frequencies of the genotypes for each cross are shown in parenthesis.

$$M = \{\mathbf{rs}^*/\mathbf{rs}, \quad \mathbf{rs}/\mathbf{ro}^*\} \tag{4}$$

epi-genotype	probability to be selected	sex
**rs*/rs**	$2(1-k-w)/(2-k-2w)$	♂
**rs/ro***	$k/(2-k-2w)$	♂
**rs*/rs***	$\rho(1-(k+w))/Z_f$	♀
**rs*/ro**	$(1-\rho)k/2Z_f$	♀
**rs*/ro***	$\rho k/Z_f$	♀
**ro/ro***	$(1-\rho)w/Z_f$	♀
**ro*/ro***	$\rho w/Z_f$	♀

Table 3: Probability of selecting an epi-genotype in the male and female pool. ρ is the probability of epigenetic silencing, k and w are the frequencies in the population of the genotypes rs/ro and ro/ro, respectively. Z_f is the total frequency of females in the population.

Using the above expression we can estimate the expected genotype frequencies in the next generation for the genotypes rs/ro and ro/ro as

$$p_{t+1} = \frac{\rho\left(3p_t^2 + 4p_t w_t - 4p_t\right) + (p_t + 4w_t)(k_t + 4w_t - 2) - 8w_t^2}{Z_t},$$

$$w_{t+1} = \frac{p_t\left(\rho(p_t+1) + 4w_t\right)}{2Z_t}, \tag{5}$$

where $Z = 4Z_f(p - 2(1-w))$. The expected difference is shown in Equation 6.

$$\Delta k = \frac{\rho\left(3p_t^2 + 4p_t w_t - 4p_t +\right) + (p_t + 4w_t)(p_t + 4w_t - 2) - 8w_t^2}{Z_t} - p_t, \tag{6}$$

$$\Delta w = \frac{k_t\left(\rho(k_t+1)+4w_t\right)}{2Z_t} - w, \tag{7}$$

The frequencies in the stationary sate can be obtained by setting Δk and Δw (Equations 6, 7) to zero and solving the system of non-linear equations:

$$\begin{cases} \frac{\rho\left(3k_t^2+4k_tw_t-4k_t+\right)+\left(k_t+4w_t\right)\left(k_t+4w_t-2\right)-8w_t^2}{Z_t} - k_t = 0 \\ \frac{k_t\left(\rho(k_t+1)+4w_t\right)}{2Z_t} - w = 0 \end{cases} \tag{8}$$

This non-linear system admits a trivial solution $O(k_0 = 0, w_0 = 0)$; to obtain the other solutions of the system we observe that it will converge to a sex ratio equal to 0.5 (in the stationary state we will have $Z_f^{ss} = Z_m^{ss} = 0.5$)

$$Z_f^{ss} = \frac{2\rho+(1-\rho)(k+2w)-1}{2} = 0, \tag{9}$$

and solving for k we obtain

$$k = \frac{1-2w(1-\rho)-2\rho}{1-\rho}; \tag{10}$$

by substituting k in the second equation of Equations 8 and solving for w we obtain two solutions:

$$w_1 = \frac{1+2\rho^3-3\rho^2-\sqrt{2(1-\rho)^3}}{2(\rho-1)^3},$$

$$w_2 = \frac{1+2\rho^3-3\rho^2+\sqrt{2(1-\rho)^3}}{2(\rho-1)^3}; \tag{11}$$

we then substitute in Equations 10, 11 to obtain

$$k_1 = \frac{2+2\rho^2-4\rho-\sqrt{2(1-\rho)^3}}{(1-\rho)^3},$$

$$k_2 = \frac{2+2\rho^2-4\rho+\sqrt{2(1-\rho)^3}}{(1-\rho)^3.} \tag{12}$$

2. Further evolution of the sex determination locus

$$\begin{aligned} q_{t+1} &= \frac{2g_tp_t\rho - g_tp_t\left(\rho-1\right)+4g_t\rho z_t+2q_tp_t\rho-q_tp_t\left(\rho-1\right)+4q_t\rho z_t}{8Z_t}, \\ p_{t+1} &= \frac{\Lambda_t\left(1-\rho\right)+p_t\left(\beta q_t+\alpha g_t\right)-4g_t\rho\left(\Theta_t\right)-4w_t\left[g_t\left(\rho-2\right)+q_t\left(2\rho-1\right)\right]}{Z_t}, \\ w_{t+1} &= \frac{\left(2g_t+p_t\left(1-\rho\right)\right)\left(p_t\left(\rho+1\right)+4w_t\right)}{16Z_t}, \\ g_{t+1} &= \frac{\left(g_t+q_t\right)\left(p_t\left(\rho+1\right)+4w_t\right)}{8Z_t}, \end{aligned} \tag{13}$$

with $Z = Z_f Z_m$, $\Lambda = r^2(3\rho+1) + 4pw(\rho+2) - 2p(2\rho+1) - 8w(1-w)$, $\Theta = (1 - g_t - q_t)$ $\alpha = 1 - 4\rho^2 + 5\rho$, and $\beta = 1 - 4\rho^2 + \rho$.

♀/♂	**rs*/rs**		**rs/ro***		**rs/xs***		**ro/xs***	
**rs*/rs***	rs/rs	(1)	rs/rs	($\frac{1}{2}$)	rs/rs	($\frac{1}{2}$)	rs/ro	($\frac{1}{2}$)
			rs/ro	($\frac{1}{2}$)	rs/xs	($\frac{1}{2}$)	rs/xs	($\frac{1}{2}$)
**rs*/ro**	rs/rs	($\frac{1}{2}$)	rs/rs	($\frac{1}{4}$)	rs/rs	($\frac{1}{4}$)	rs/ro	($\frac{1}{4}$)
	rs/ro	($\frac{1}{2}$)	rs/ro	($\frac{1}{2}$)	rs/xs	($\frac{1}{4}$)	rs/xs	($\frac{1}{4}$)
			ro/ro	($\frac{1}{4}$)	rs/ro	($\frac{1}{4}$)	ro/ro	($\frac{1}{4}$)
					ro/xs	($\frac{1}{4}$)	ro/xs	($\frac{1}{4}$)
**rs*/ro***	rs/rs	($\frac{1}{2}$)	rs/rs	($\frac{1}{4}$)	rs/rs	($\frac{1}{4}$)	rs/ro	($\frac{1}{4}$)
	rs/ro	($\frac{1}{2}$)	rs/ro	($\frac{1}{2}$)	rs/xs	($\frac{1}{4}$)	ps/xs	($\frac{1}{4}$)
			ro/ro	($\frac{1}{4}$)	rs/ro	($\frac{1}{4}$)	ro/ro	($\frac{1}{4}$)
					ro/xs	($\frac{1}{4}$)	ro/xs	($\frac{1}{4}$)
**ro*/ro**	rs/ro	(1)	rs/ro	($\frac{1}{2}$)	rs/ro	($\frac{1}{2}$)	ro/ro	($\frac{1}{2}$)
			ro/ro	($\frac{1}{2}$)	ro/xs	($\frac{1}{2}$)	ro/xs	($\frac{1}{2}$)
**ro*/ro***	rs/ro	(1)	rs/ro	($\frac{1}{2}$)	rs/ro	($\frac{1}{2}$)	ro/ro	($\frac{1}{2}$)
			ro/ro	($\frac{1}{2}$)	po/xs	($\frac{1}{2}$)	ro/xs	($\frac{1}{2}$)

Table 4: Possible crosses. The expected frequencies of the genotypes for each cross are shown in parenthesis.
